# Considering the response in addition to the challenge – a narrative review in appraisal of a motor reserve framework

**DOI:** 10.18632/aging.205667

**Published:** 2024-03-18

**Authors:** Daniel Zeller, Shawn Hiew, Thorsten Odorfer, Carine Nguemeni

**Affiliations:** 1 Department of Neurology, University Hospital Würzburg, Würzburg 97080, Germany

**Keywords:** motor reserve, cognitive reserve, aging, compensation, neurodegenerative disease

## Abstract

The remarkable increase in human life expectancy over the past century has been achieved at the expense of the risk of age-related impairment and disease. Neurodegeneration, be it part of normal aging or due to neurodegenerative disorders, is characterized by loss of specific neuronal populations, leading to increasing clinical impairment. The individual course may be described as balance between aging- or disease-related pathology and intrinsic mechanisms of adaptation. There is plenty of evidence that the human brain is provided with exhaustible resources to maintain function in the face of adverse conditions. While a reserve concept has mainly been coined in cognitive neuroscience, emerging evidence suggests similar mechanisms to underlie individual differences of adaptive capacity within the motor system.

In this narrative review, we summarize what has been proposed to date about a motor reserve (mR) framework. We present current evidence from research in aging subjects and people with neurological conditions, followed by a description of what is known about potential neuronal substrates of mR so far. As there is no gold standard of mR quantification, we outline current approaches which describe various indicators of mR. We conclude by sketching out potential future directions of research.

Expediting our understanding of differences in individual motor resilience towards aging and disease will eventually contribute to new, individually tailored therapeutic strategies. Provided early diagnosis, enhancing the individual mR may be suited to postpone disease onset by years and may be an efficacious contribution towards healthy aging, with an increased quality of life for the elderly.

## INTRODUCTION

In its common use, the term “reserve” refers to something stored or kept available for future use or need. In physiology, the capability of an organ to carry out its activity under stress is known as physiologic reserve [1]. There is plenty of evidence that the human brain is provided with similar resources to maintain function in the face of adverse conditions [2–4]. Indeed, frequent discrepancies between a person’s underlying level of brain pathology and the observed functional deficits are commonly attributed to individual degrees of reserve [5, 6]. In this context, the individual neurobiological capital in terms of quantifiable brain properties like number of neurons, synapses, or gray matter volume is captured by the term of brain reserve (BR). Inter-individual variation in structural characteristics of the brain may then explain differential susceptibility to functional impairment in the presence of pathology or other neurological insult [3, 7]. Apart from the rather “hardwired” concept of BR, the term of neural reserve refers to individual differences in the efficiency of networks which are also commonly used by unimpaired subjects or to the use of alternative strategies for task performance [8]. Complementary, neural compensation comprises the utilization of alternative networks not typically used by healthy individuals in order to maintain or improve performance of a task [8]. As a general rule, neither of these mechanisms provides protection against the accumulation of brain pathology itself, but rather mitigates its negative consequences. Hence, retained brain function despite intrinsic or extrinsic interference may indicate a high reserve, while occurrence of tangible dysfunction may mark the exhaustion of reserves. To date, the “reserve concept” has mainly been coined in the field of cognitive neuroscience. However, emerging evidence suggests similar mechanisms to underly individual differences of the adaptive capacity within the motor system [9–13]. In this review, starting from a short outline of the cognitive reserve concept and how it has been approached, we summarize what has been proposed so far about a reserve equivalent in the motor system. We conclude by sketching out potential future directions of research.

## Cognitive reserve – pioneering a general principle?

The idea that cognitive performance not only depends on brain pathology but also may be influenced by some kind of intrinsic properties of the individual subject’s brain originates from the 1980s: In a postmortem examination, a group of subjects who had suffered from mild Alzheimer’s disease presented with higher brain weights and greater numbers of neurons as compared to age-matched control subjects [5]. The authors speculated that these people may have had incipient Alzheimer’s disease but escaped loss of large neurons or may have started with larger brains and a higher number of large neurons in terms of a greater reserve [5]. In the meantime, the term “cognitive reserve” (cR) is widely used, and a large body of literature has addressed its relationship to education and other lifetime experiences as well as its role in ageing and neurodegeneration (reviewed in [14]). According to Yaakov Stern who is prominent exponent of the framework, cR refers to an active model where the threshold for functional decline is determined by both quantitative brain measures, and life experiences [14, 15]. The NIH collaboratory workgroup on cognitive reserve and resilience defined cR as a property of the brain that allows for cognitive performance that is better than expected given the degree of life-course related brain changes and brain injury or disease [16]. A number of proxies, single or in combination, has been used to estimate the individual cR. Common (predominantly biographical) measures are number of years of formal education, estimated premorbid intelligence quotient (IQ), measures of lifetime occupation and participation in cognitively stimulating activities, participation in leisure activities, physical activity and exercise, and social engagement [7, 17]. Those factors are thought to increase brain plasticity and resistance to cellular death and other age-related phenomena (e.g., synaptic and white matter changes, pathological modifications, etc) [18, 19].

**Figure 1:**
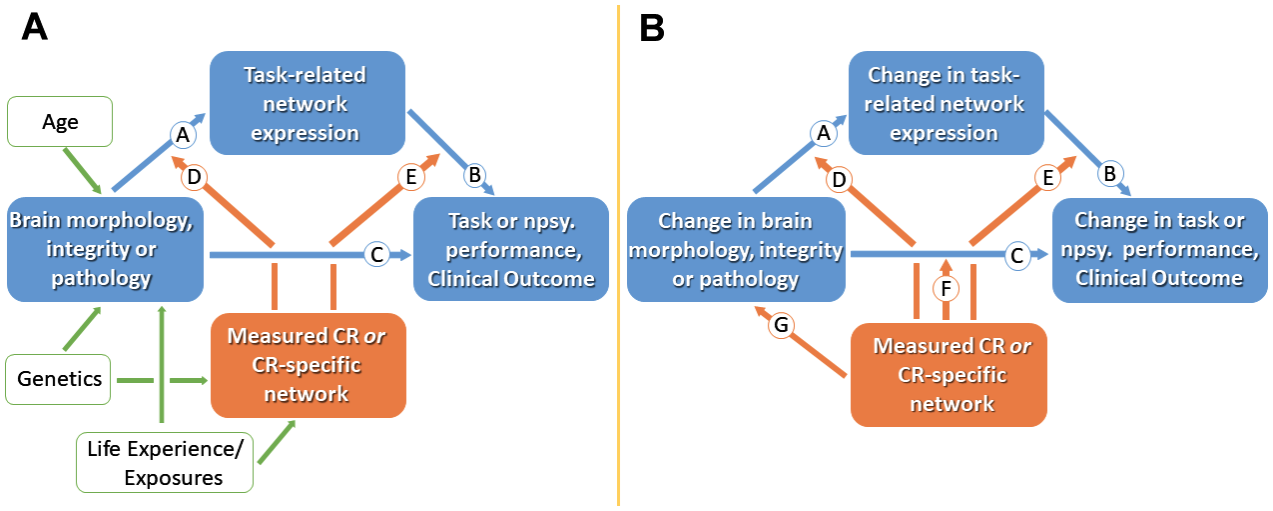
Cross-sectional (**A**) and longitudinal (**B**) models for studying cognitive reserve (CR). (**A**) Measures of brain morphology, integrity or pathology may impact clinical status via path Ⓒ. CR is represented by the orange box; working measures of CR include CR proxies or identified CR brain networks. Age, genetics and life experiences are believed to influence brain measures and CR. CR is assumed to moderate the effect of brain status on clinical status, thus producing individual differences in the clinical correlates of a given level of brain reserve and brain pathology. The effect of brain status on clinical status may be mediated in part by brain networks captured during task related activation (paths Ⓐ and Ⓑ). Path Ⓓ suggest that CR might moderate between brain status and activation. Path Ⓔ recognizes that some aspects of CR might moderate between brain and clinical function without being captured in specific task-related activations. (**B**) In longitudinal models, two paths can be added: path Ⓕ assesses how CR moderates the effect of brain change on cognitive change, and path Ⓖ addresses neuroprotective mechanisms. Npsy., neuropsychological. Adopted from [7].

Effectively, two approaches have been described to assess the impact of cR: a cross-sectional and a longitudinal research scheme [7]. Following the cross-sectional approach, the parameters brain status, task performance, measured cR (by questionnaire), and (optionally) task-related network expression are assessed once in a selected group. Then, cR is posited to moderate the relationship between the brain status and task performance, a hypothesis that might be tested by stratification or correlation (Figure 1A). For instance, given a certain gray matter volume, subjects with higher cR perform better in cognitive tasks [20], most likely indicating that they make “better use” of the available neural resources [7]. Concomitantly, individuals with high cR show increased network efficiency as evidenced by functional imaging [21, 22].

However, looking for a parameter which can determine the preservation of function over time, a longitudinal approach may be more suitable [23]. The latter implies a prospective design with at least one follow-up assessment of the dependent parameters, i.e. brain status and task performance, allowing the calculation of changes of these parameters along the time axis (Figure 1B), therefore studying “reserve in action”. As such, longitudinal studies may help establish causal relationships, which is a clear advantage as compared to cross-sectional designs.

As a third attempt to directly measure cR, several authors have used a residual approach: When the variance in memory performance has been accounted for by brain measures and demographics, the remaining variance showed high correlation with common cR proxies and predicted subsequent cognitive decline [7]. Similarly, the difference between the chronological age predicted by gray matter atrophy and actual age of a subject is associated with CR measures [7].

## Motor reserve – general prerequisites

Discrepancies between apparent pathology and actual functional level are not restricted to the cognitive domain. Indeed, the predictive value of a given pathology (whether histological or radiological) for motor function may also be rather weak, as has been demonstrated in neurological conditions like multiple sclerosis or Parkinson’s disease [13, 24–27]. This suggests a modificatory parameter which, along the lines of the cognitive terminology, may be referred to as motor reserve (mR). Similar to cR, mR may be viewed as a framework where the threshold for functional (motor) decline is determined by lifetime motor (and probably cognitive) activities and other environmental factors that explain differential susceptibility to functional impairment in the presence of pathology [8]. In other words, the mR may constitute a capital of brain structural and functional alterations throughout life that can provide resilience (i.e. the ability to cope with brain pathology [13]) to age- or disease-associated motor skill decline. Thus, other terms coined in the context of cR may be helpful to describe key properties of a mR framework (Table 1).

**Table 1:** Glossary of terms related to the motor reserve as transferred from the concept of cognitive reserve.

	**Motor reserve (mR)**	**Cognitive reserve (cR)**
	mR mitigates **motor decline** by adaptation of **motor-relevant** networks. Current evidence points to more efficient and integrated use of distinct functional networks.	cR mitigates **cognitive decline** by changes in **cognition-relevant** networks. These changes can for example involve network reorganization or more efficient use.
Brain Reserve	Differences in brain size and other quantitative aspects of the brain that explain differential susceptibility to functional impairment in the presence of pathology or other neurological insult. Brain reserve reflects the neurobiological capital of the brain as a fix construct (numbers of neurons, synapses, grey matter volume etc.) at any point in time, not involving active adaptive or functional processes in the face of injury or disease.	
Neural Reserve	One proposed neural basis of reserve. It involves **motor** or **cognitive** networks which are used by unimpaired individuals (as opposed to the use of alternative networks). Individual differences in network efficiency/capacity or the use of alternative strategies within the same network may provide reserve against the impact of brain changes.	
Neural Compensation	One proposed neural basis of reserve involving the utilization of alternative networks not typically used by healthy individuals in order to maintain or improve **motor** or **cognitive** performance.	
Network Efficiency	The degree to which a task-related brain network must become activated in order to accomplish a given task.	
Physical Resilience	A characteristic which determines an individual’s ability to resist or recover from functional decline following health stressors.	

(derived from [8], and from [13, 16, 36]).

**Figure 2:**
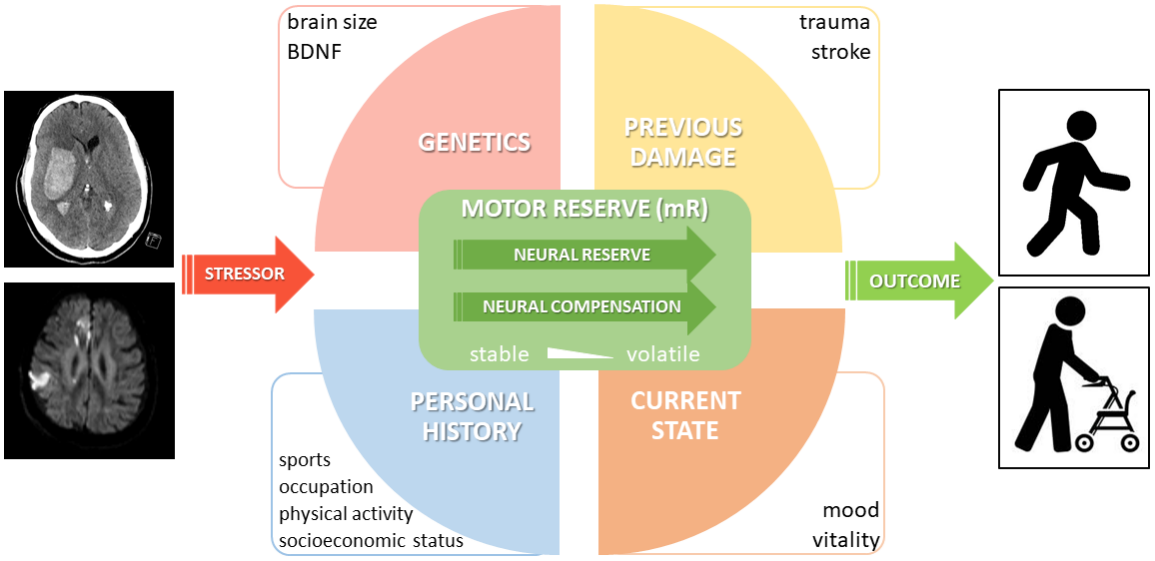
**Proposal of a conceptual model of motor reserve (mR).** A selection of important parameters with an impact on mR is shown. While neural reserve and (beneficial adaptive) neural compensation are considered part of the proposed neural basis of mR, it should be noted that neural compensation may only be related to reserve if it is linked to a third (e.g. lifestyle) factor and shows individual variation.

Because of the novelty of this concept, literature on the neurobiological underpinnings of mR is still relatively scarce. Yet, available evidence points to a number of (substantially interacting) intrinsic and extrinsic parameters that could account for the current quantum of mR. The existence and the interaction between those parameters might set a critical threshold determining the amount of perturbance that can be borne without persistent functional impairment (Figure 2). These parameters unfold their effects on rather different time scales. Genetic contributors, which may, for instance, define brain size and architecture or polymorphisms, can be considered rather stable over time, thus entailing “trait-like” properties and susceptibilities of a particular person to stressors [28, 29]. At the volatile end of the scale, there are mR fluctuations according to the current state of a person, like present vitality or mood. Moreover, the individual history of physical activity, i.e., sports, training, occupation (sedentary vs. physical occupation) is believed to be an important modulator of neuroplasticity, an important substrate of mR [30]. Most likely, this includes stable aspects if an elderly person performed training on a regular basis for decades, but may also be susceptible to increased physical activity within previous days or weeks [31, 32]. This factor suggests social inequality might be at play such that individuals with lower socioeconomic statuses are especially vulnerable to accelerated aging and increased gait impairment due to poorer access to stimulating environments and facilities for physical activity [33, 34]. Better understanding the link between lifelong physical activity and age- and disease-related decline may help us take steps towards reducing social inequality in access to physical activity and protect aging adults from all walks of life. Additionally, previous stressor experiences might already have challenged mR, eventually leaving the subject with reduced or, in theory, even with increased mR capacity, probably similar to the plasticity which has been described for the limbic network [35].

At a given timepoint, impaction by a defined stressor would result in a certain motor outcome, the favorability of which is determined by current mR.

## What may be the substrate of motor reserve?

**Figure 3:**
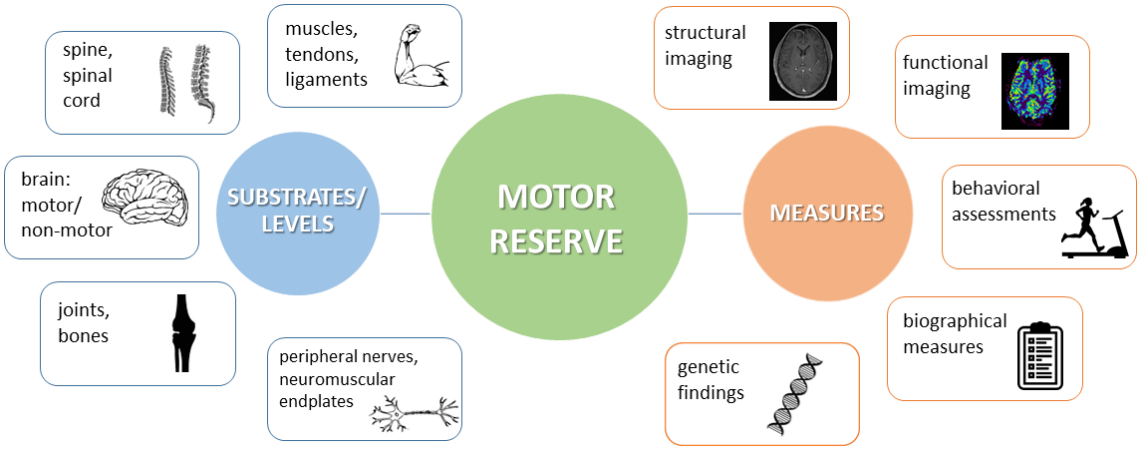
Schematic view on the different substrates and levels where motor reserve (mR) might be located (left panel) and on examples of how to measure mR (right panel).

Human motor activity comprises an interplay of neurological and musculoskeletal, central and peripheral, motor and non-motor aspects, all of which make their contribution to the overall motor capacity. Professional athletes or musicians impressively exemplify the high levels of motor performance which can be reached owing to continuous training efforts. Along the same lines, in response to perturbation, physiological mechanisms at each level may spring into action in order to support restoration of function (Figure 3, left panel).

No finite boundary exists between the central and peripheral components of mR and endurance [37]. Under stress, each component (e.g., brain, nerves, muscles, skeleton) may provide function within its limits. In a neuroscientific sense, however, the term “motor reserve” refers to the set of alterations in the brain at structural and functional levels throughout one’s life that may provide resilience against neuropathology-associated functional decline [12]. These alterations do not necessarily take place in the afflicted (e.g., degenerating) brain regions. Rather, they may take place in regions located upstream or downstream of the interference, in the connectivity between disturbed areas, or in relatively independent structures [12]. Within this CNS-centered perspective, it appears consequential to regard the entirety of peripheral (including neuromuscular and musculoskeletal) impairment as to challenge mR and to ask for compensation. Consistently, neurological conditions affecting the CNS would represent an internal challenge of mR, while all other conditions would be summarized as external ones.

**Figure 4:**
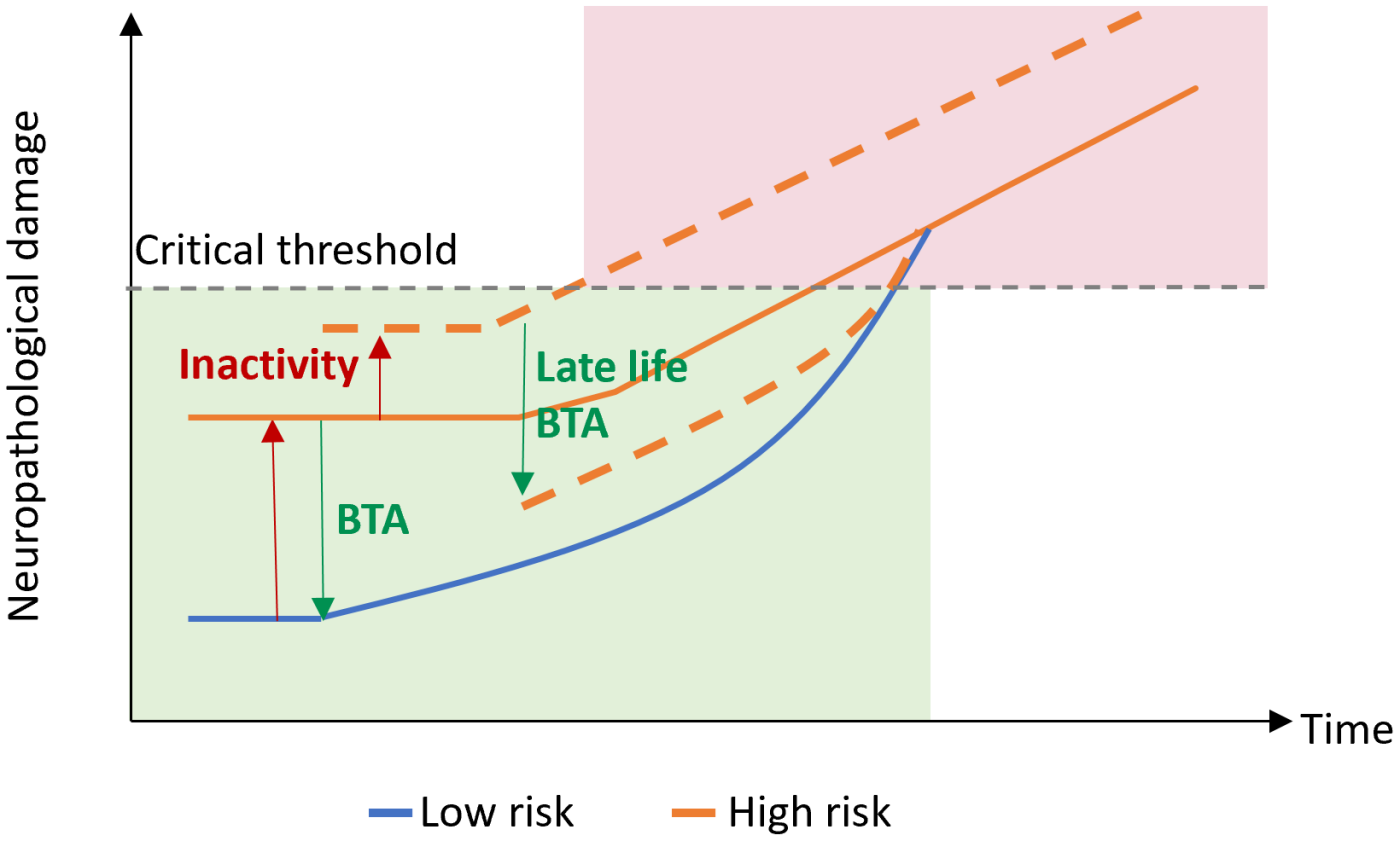
**Motor reserve model.** Motor reserve accounts for normal motor function (green) until neuropathological damage associated with aging and/or neurodegenerative disease reaches a critical threshold, after which impaired motor function may be observed (red). BTA, beneficial training activity.

Conceptually, mR accounts for normal motor performance (on the behavioral level) until interference reaches a critical threshold (Figure 4). This comprises a passive process incorporating network redundancy and an active reserve based on an extended recruitment of normal networks and additional recruitment of novel areas [38]. Implicating current concepts in the field of cognitive reserve, the traditional separation of cR as the active (“software”) and brain reserve as the passive (anatomical, “hardware”) part of resilience has gradually been abandoned [7, 39]. Since life experiences as well as exercise have been shown to support maintenance of brain integrity and to modulate regional brain volumes [40], the dichotomy might better be transferred into an integrative view, where brain regions, their projections and synaptic interactions are part of a motor network [41]. Within this framework, mR would be mirrored in the particular network’s capacity to process information efficiently [41]. Such efficiency may rely on both structural and functional properties of the CNS, both of which are subject to use- and experience-dependent neuroplastic changes. Neuroplasticity refers to the brain’s ability to adapt to changes by modifying neural connectivity, brain function, and ultimately brain structure in response to changing demands and environments throughout the lifespan [42–44]. Depending on the particular challenge, i.e., whether a person is learning, aging, or recovering from a neurological condition, it may subserve the gain, maintenance or restoration of brain function. From the functional perspective, neuroplasticity may be considered beneficial if function is improved or regained [45].

## Motor reserve – current evidence

Published articles containing the fixed search term “motor reserve” are summarized in Supplementary Table 1. They served as entry points to summarize current evidence of mR in a narrative fashion, along the subtopics of aging, neurodegenerative disease, stroke and concussion. While all these conditions are suited to pose a challenge to the individual mR, it is important to bear in mind that there are considerable differences which relate to their spatial and pathophysiological, but in particular to their temporal properties. For example, PD is a neurodegenerative disease predominantly characterized by a slowly progressive loss of dopaminergic terminals, but eventually affecting most of the brain. Aging might even rank at the slower end of progression speed. In contrast, stroke is an event that commonly occurs focally and acutely at a single point in time. Thus, the underlying mechanisms of mR and their dynamics may show high variability: Slower and more distributed processes are likely to trigger adaptive steps (at least initially) keeping pace with the pathological condition, whereas acute events would overburden the adaptive capacity and cause temporary or permanent impairment [2, 6].

### Evidence for mR in aging

In a seminal piece of work on motor function in the elderly, Elbaz and colleagues demonstrated that more-educated persons are less susceptible to the effect of white matter lesions on motor function [46]. While higher education was associated with better motor performance, there was no association with slower decline. This supports a model of “passive reserve”, i.e., the persistence of education-related differences in motor performance over time, rather than less pronounced decline due to active compensation [46].

While the individual education is not accessible for retroactive modification, physical activity is a modifiable behavior at any age [47]. Earlier animal and human studies have already suggested that physical activity may mitigate the consequences of accumulating brain pathology on motor function [48–50]. Indeed, in older adults without dementia, high levels of physical activity – as assessed by wearable actigraphs for up to eleven days – were shown to reduce the effect of white matter hyperintensity burden on motor function [47]. In persons with the highest physical activity, white matter hyperintensities did not affect motor function at all, whereas there was a strong negative association in less active people [47]. Thus, higher levels of physical activity may amplify the individual mR. While the underlying neural mechanisms still remain hypothetical, production of growth factors such as BDNF, FGF-2, IGF-1, and VEGF might not only enhance tissue protection, but might also promote synapto- and neurogenesis as potential contributors to brain reserve [47, 49].

Physical activity is known to positively affect cognition in older adults. The total amount of physical activity carried out throughout an individual’s lifespan may have an even more pronounced impact on cognition in old age. In a group of 75 healthy individuals over 50 years of age, global cognitive function was strongly related to measures of current and lifetime physical activity. Thus, physical activity training could be an important component of preventive interventions [51].

Normal aging is believed to reduce the efficiency of brain networks in terms of a decreasing neural reserve [52, 53]. Subclinical alterations may be unmasked by dual-task (DT) conditions during walking [54]. In the elderly, DT decreases gait speed and swing time while increasing swing time variability. These DT effects are related to individual walking abilities as such, executive function, and mood, but can partly be attributed to limited cognitive and motor reserves [54]. At least for cognitive paradigms, formal education is known to mitigate DT effects, thus lending support to the use of DT as an approach to reserve [55, 56].

### Evidence for mR in neurodegenerative disease

Age-associated neurodegenerative diseases, including Parkinson’s disease (PD) and amyotrophic lateral sclerosis (ALS), typically present with region-specific neurodegeneration [57]. Neuronal Lewy bodies, PD’s primary pathological hallmark, are earliest detected in the medulla oblongata and olfactory bulb, while subsequent pathological involvement of the midbrain is associated with classical parkinsonian motor symptoms (bradykinesia, rigidity, resting tremor) [58]. ALS is a rapidly progressive neurodegenerative disease, characterized clinically by the presence of both upper and lower motor neuron involvement. Most patients possess neuronal inclusions positive for the RNA binding protein TDP-43, which are noted in the ventral spinal cord, lower brainstem and frontal cortex [59]. A large body of evidence indicates that the clinical manifestation of neurodegenerative diseases is preceded by a preclinical phase for years if not decades [60, 61]. These prodromal stages are characterized by typical pathological and/or imaging findings along with a lack of motor symptoms and therefore suggest effective compensatory mechanisms. Postponing the point of clinical manifestation would be an attractive target for future therapies. This similarly applies to neurodegenerative diseases that are not primarily age-related but are monogenetic and have heterogeneous ages of clinical onset, such as spinocerebellar ataxia type 2 (SCA2) or Huntington’s disease (HD).

### Parkinson’s disease

The majority of studies assessing mR in the context of neurodegenerative diseases till date include persons with PD.

Based on MRI volumetric data, estimates of the individual mR have been shown to correlate with local striatal volumes (LSV) of the bilateral caudate, anterior putamen, and ventro-posterior putamen, suggesting LSV as a neural correlate of brain reserve as part of the mR in PD [13, 62]. Notably, large LSV was associated with low initial L-dopa equivalent doses (LED), but accelerated LED increment later [62]. This finding might be in favor of an active mR model where the rate of motor symptom progression is different depending on the disease stage [62].

By means of diffusion tensor imaging (DTI), a recent study identified a number of additional (extra-basal ganglia) brain structures related to mR estimates in PD [63]. DTI is an MRI technique which can detect microstructural changes within neural tissue. Estimates of mR were mainly associated with neuronal fiber integrity of frontal and temporal lobes, limbic structures, nucleus accumbens, and thalamus, indicating that DTI may capture certain aspects of brain reserve which might account for individual differences in motor deficits in PD despite similar pathologic changes.

Sequencing the glucocerebrosidase (GBA) gene in 408 PD patients, the same group aimed to investigate GBA variants as potential biomarkers of mR [64]. Compared to PD patients without mutations, those with GBA mutations were younger, but had higher Unified Parkinson’s Disease Rating Scale (UPDRS) motor scores for the less affected side despite comparable DAT signals, thus indicating a reduced capacity to cope with PD-related pathology [64]. While this finding may underline the basic influence of genetics on mR estimates, it does not allow the attribution to specific underlying mechanisms.

Applying a sinusoidal force production task during fMRI in PD patients and matched controls, Palmer et al. found controls to recruit additional resources with increasing task difficulty. Patients in the OFF-medication state showed a higher extent of recruitment of the normal network (referring to “neural reserve”) and recruited additional regions (referring to “neural compensation”) in comparison to ON-medication. This may represent a correlate of mR allowing PD patients at early stages to maintain near to normal motor output [38]. The amount of mR may also be enhanced by premorbid physical activity in people with PD. Indeed, when matched for dopamine transporter activity in the posterior putamen, PD patients within the highest tertile of premorbid physical activity presented with lower UPDRS motor scores compared to patients of the lowest tertile. Thus, premorbid exercise may act as a proxy for an active mR in PD [65]. In their follow-up study over three years, the same authors found that higher baseline mR estimates were associated with a lower risk of developing L-dopa induced dyskinesias (LID) and freezing of gait (FOG), and lower longitudinal LED increases. Thus, mR seemed to be maintained with disease progression and may modulate risks of LID and FOG [66].

In PD animal models, enhanced physical and cognitive stimulation can reduce motor deficits [67]. Based on stratification of 182 PD patients with similar disease duration by years of education (high vs low education), patients with >12 years of education scored higher in the Mini–mental state examination and showed fewer motor deficits [68]. This confirms earlier findings that a low number of years of education is associated with high UPDRS motor scores in PD patients, suggesting a protective effect of higher educational attainment on motor function [69].

In 102 PD patients considered for DBS surgery, age and level of education were correlated with UPDRS in the OFF-medication state. At follow-up two years after surgery, UPDRS-ON was correlated with level of education and cognitive state, i.e., patients with higher education presented with better motor outcome post DBS [70]. Unlike what is known from cR so far, a putative enhancing effect of education on mR was still evident 12 years after onset of PD symptoms. Intensive education may increase synaptic plasticity, effectiveness of brain networks, and regeneration mechanisms, but may also have indirect effects on lifestyle or socioeconomic status, all of which have directly or indirectly been linked to mR [70].

While physical activity appears to implicate lower PD risk, one might speculate that in those with high mR, the diagnosis of PD could be delayed. Aiming to disentangle containment of brain pathology from greater mR capacity, a longitudinal study in the Vasaloppet population, i.e., a large subgroup within the Swedish patient registry which participates in a long-distance cross-country ski race, reported lower incidence of PD among skiers. The association dissipates with time and is consistent with a greater mR. In other words, skiers appear to have similar brain pathology but take longer to develop clinical PD [71].

A recent study applied resting state fMRI to evaluate the functional brain network associated with mR in early PD [72]. Based on a residual model, the major hubs of the network were located to the basal ganglia, inferior frontal cortex, insula, and vermis. Insinuating the model of neural reserve, greater connectivity within this network was associated with greater mR and slower increase of LED over time [72].

A history of cancer has been discussed to enhance mR in PD patients. Indeed, comparison between PD patients with no prior neoplasia, with premobid precancerous condition, and with premorbid malignant cancer revealed lower motor impairment despite similar levels of dopamine depletion in patients with premorbid cancer [73]. While such associations may reveal important drivers of general resilience, they do not provide any mechanistic insights as to how particular aspects of mR are related to the medical history of cancer.

Threshold-free network-based statistics on MRI data on 238 drug-naïve PD patients indicated a mR-associated structural network with nodes mainly in the frontal region and the cerebellum. As a potential indicator of neural reserve, higher strength of this network was associated with a decelerated LED increase during a 3-year follow-up period [74].

It appears likely that cognition and mR need to be considered interacting rather than discrete phenomena. Accordingly, an estimate of mR based on initial motor deficits and striatal dopamine depletion (residual model) was found to correlate with verbal memory function, years of education, and white matter integrity in the left fornix in 163 drug-naïve PD patients [75]. Conversely, higher mR estimates tended to be associated with a lower risk of dementia conversion [75].

Altogether, evidence of mR in PD is multifaceted, with impact by genetics (brain volume, GBA variants) and premorbid experiences (physical activity, education, comorbidities), with a reciprocal relationship between mR and cR, and with a number of potential surrogates predicated on structural and functional MR imaging (cf. [13, 76]).

### Amyotrophic lateral sclerosis

While cognitive reserve has just started to receive some attention in people with ALS [77], data on mR are scarce. Accordingly, in a “perspective” on ALS, Bede et al. point to the lack of consideration of compensatory processes in existing studies, which exclusively focus on degenerative changes [6]. Actually, fMRI studies using motor paradigms in ALS patients have consistently shown an activation shift from the primary motor cortex to premotor, supplementary motor, ipsilateral motor, basal ganglia, and cerebellar regions, thus suggesting neural compensation [6, 78]. Resting state EEG studies in persons with ALS have identified increased connectivity in the default mode and corticocerebellar networks, probably indicating neural reserve [79]. In addition, increased cortical volumes in supplementary motor regions (as supposable correlates of brain reserve) have been shown in ALS compared to controls [80].

Taken together, these few observations in ALS may comprise typical signatures of plastic changes like those which have already been linked to mR in other neurological conditions, and thus deserve closer attention in future research.

### Spinocerebellar ataxia type 2

Taking the Motor Reserve Index Questionnaire (MRIq) as a measure of mR in 12 patients with SCA2, Siciliano and coworkers found an association of the MRIq with the severity of motor symptoms, educational and intellectual levels, and executive functions. Moreover, they found a pattern of MRI functional connectivity within subnetworks of specific cerebellar and cerebral areas which was associated with MRIq scores. The authors suggest this network being a potential biomarker of mR which may be influenced by education and cognitive function [81].

### Huntington’s disease

Physical activity has also been found to contribute to favorable clinical outcome parameters in HD [82]. A positive influence of higher physical activity, as measured by means of wearables, on motor function (Symbol Digit Modalities Test) and daily life (World Health Organization Disability Assessment Schedule) has been demonstrated for the prodromal and early stages of HD [82].

### Evidence for mR in other neurological conditions

Stroke is a main cause of disability and a major cost factor for health care systems. Accordingly, lowering stroke-related impairment and enhancing functional recovery is of outstanding interest, and it seems timely to gather knowledge about the significance of individual reserve measures in this context. While a number of studies aimed to identify predictors of recovery after acute stroke (e.g. [83, 84]), others assessed particular aspects of cR [85, 86] or mR (e.g. [87, 88]) and their potential influence on recovery.

In the special case of CADASIL patients, the shape of the central sulcus, more specifically the vertical position of the hand knob, was strongly associated with overall clinical function as rated by the modified ranking scale, suggesting a simple indicator of brain reserve and associated mR [89].

Sport-related concussion is another example of an acute impact on CNS integrity. Immediately after the acute trauma, cognitive-motor integration is impaired as evidenced by a visuomotor transformation task. Following-up task performance after concussion, subjects with high sport experience (≥7 years) reach a normal performance level quicker than those with low sport experience. This may indicate higher mR in youth with greater sport experience [90].

## How best can the motor reserve be quantified?

Several biological traits (e.g., brain size) and sociodemographic factors (e.g., years of education) have been associated with cR [2]. Given the complexity of the venture to measure reserve, some authors considered cR a “hidden variable” [91] which cannot be directly observed but inferred by observation of other variables [2]. As observed in the clinical studies previously mentioned, it is more than likely that the same applies to the construct of mR, and indeed, there is no gold standard for a global assessment and quantification of mR so far. In the following, we outline current approaches describing different facets (“indicators”) of mR (Figure 3, right panel):

Structural properties of the brain may be assessed autoptically or by means of magnetic resonance imaging (MRI). While post-mortem findings of regional increase or decrease of neuronal density may allow for cross-sectional correlation analyses of motor function and reserve and might support retrospective evaluation of lifestyle interventions, they are naturally unsuitable for prospective approaches [12]. The same applies to proteomics in postmortem brain samples [92]. Therefore, MRI-based morphometry is the method of choice to assess hyper- or hypotrophy of brain regions. For instance, cortical thickness of the motor cortex has been correlated with past physical activity or occupational history [40], and regional reduction of gray matter volume was associated with measures of physical frailty [93]. Diffusion tensor imaging (DTI), an estimate of structural connectivity, revealed shorter path length and higher global efficiency of brain networks in high-level basketball players as compared to controls [94]. One hour of neurofeedback training with motor imagery was associated with increased fractional anisotropy in the sensorimotor segment of the corpus callosum [95]. Learning a complex visuomotor sequence over five consecutive days came along with changes of WM microstructure in the tracts underlying the sensorimotor cortices [96].

In functional imaging, conclusions on brain and network function are drawn from changes in metabolism, blood flow, or absorption. Task-related fMRI highlights those brain regions which are involved in a specific task. In normal aging, more widespread recruitment of brain regions for a particular task suggests that more resources are required to achieve the same goal as individuals age [97]. In this context, mR may modulate network efficiency, for example by shifting the ceiling so that, given the same neural resources, greater magnitudes of learning and/or recovery can be achieved. Conversely, victims of nonfatal drowning who performed a finger tapping task during fMRI acquisition showed an increased brain response in the left putamen and insula compared to controls, probably indicating higher “demand” due to reduced brain reserve [98]. With increasing difficulty of a force production task during fMRI, PD patients recruit additional resources as compared to matched controls, and patients OFF-medication recruit the normal motor network more extensively, along with additional regions [38]. Based on a residual model in people with early PD, resting state fMRI revealed a network between the basal ganglia, inferior frontal cortex, insula, and vermis, where greater connectivity was associated with greater mR [72]. Other studies used Dynamic Causal Modeling [99] or time-resolved functional connectivity [100] to estimate compensatory mechanisms and mR.

At the behavioral level, various motor learning and adaptation paradigms might be suitable and have been used to probe motor capacity and reserve [101, 102]. Applying stepwise increment of task difficulty and dual task paradigms are options to max out the individual mR and to define its limit [55, 103, 104]. Especially if motor performance is unimpaired, it cannot be discriminated whether mR is still untouched or if performance is kept normal at the expense of mR. Interventional approaches using non-invasive brain stimulation and/or motor training paradigms to disrupt or enhance underlying mR networks, respectively, can be useful in establishing causal relationships [105–107].

In analogy with cR, biographical measures of prior physical activity, exercise, and occupation across defined time spans may be well suited to estimate individual mR [65, 70, 71, 108, 109]. The application of questionnaires is easy and inexpensive and can be extended to virtually all fields of everyday physical activity, though it might be limited by the individual ability of retrospection.

In future, genetic findings, e.g., BDNF polymorphisms, might provide valuable information about specific individual aspects of mR [110].

Similar to cR, the role of mR may be studied with cross-sectional, residual, or longitudinal approaches (Figure 1). In general, by drawing one sample at a single point in time, it might be demanding if not impossible to reliably deduce the particular contribution of mR to motor performance at this timepoint. If baseline performance (prior to aging or disease) is unknown, the large interindividual variability of motor trajectories may prevent clear individual conclusions. Thus, cross-sectional studies prove useful to understanding relationships on the (large) group level. However, if one wants to assess mR as an individual characteristic which may be the target of an individually tailored mR enhancement, residual or longitudinal approaches, most likely based on a composite measure estimating current mR, seem much more promising.

The search for the best surrogates of mR has only just started, and the main challenges of this venture have been outlined concisely in a current viewpoint on the mR framework in PD [13]. Referring to current post-mortem evidence in humans [111] and previous rodent studies, the authors subsume that lifetime factors may contribute to the building up of resilience mechanisms such as lower neuroinflammatory response and greater neuronal substrate, which in turn may foster network adaptations against motor decline [13]. As an important caveat, the authors recommend investigating the interindividual variability of underlying neuronal mechanisms of mR rather than the pure behavioral output in order to assess the role of modifiable and nonmodifiable factors on mR [13]. In addition, they point to the assumably strong relationship between cR and mR which should implicate an assessment of both domains in all studies dealing with measures of reserve. Moreover, the authors emphasize that compensatory mechanisms might just reflect common responses to brain damage, but not necessarily be a measure of the individual capacity of resilience. The crucial step will be to examine the mitigating effect of different resilience signatures in longitudinal designs to estimate their power in slowing the disease course [13]. Indeed, in our opinion, there are good reasons to consider cross-sectional assessments inferior to longitudinal approaches, where a set of prospective mR estimators may be probed for their predictive value with respect to individual trajectories of a disease course or aging.

It is however important to keep in mind that a discrimination between cognitive and motor reserve might be challenging as they both rely on the activation of crossed neural networks with common substrates that may be difficult to probe individually. In the light of their strong reciprocity, it even can be advocated for a more general approach to reserve as it may have already earlier been implicated by concepts like the “motoric cognitive risk syndrome” [112]. Exemplarily, current neurobiological evidence regarding resilience in PD points to an involvement of brain areas related to motor function, but also to motivational and planning domains [13]. Taking into consideration that PD, as many other neurological conditions, comprises both motor and nonmotor symptoms, an isolated view of mR may not reflect the entire spectrum of resilience towards the pathophysiology of PD [13].

On the other hand, even if there was an accepted quantitative measure of global mR, it would be likely for two individuals with the same "total amount" of mR to be differentially susceptible to domain- or task-specific challenges. Thus, when it comes to the development of targeted interventions aiming at an enhancement of mR, it might be necessary to focus on domain- or even subdomain-specific measures.

Within this ambivalence, we consider it an important first step to understand basic properties and estimators of mR before time has come for an overarching framework of reserve.

## Why motor reserve? – future directions

Human life expectancy has been increasing at a rapid rate. However, this dramatic increase in life expectancy did not come with a proportionate increase in quality of life for the elderly [113]. Generally, the risk of disease, disability, dementia and advanced aging prior to death has increased. In particular, aging is associated with an increasing incidence of neurodegenerative disorders which are characterized by loss of specific neuronal populations along with increasing clinical impairment. Despite decades of intensive research, curative treatments are still lacking.

The individual disease course may be described as the balance between disease-related pathology and intrinsic mechanisms of adaptation. With increasing evidence pointing to a key role of intrinsic compensation mechanisms, strategical considerations for the development of new therapies aim at an early modulation of the disease course on the patients’ side. Provided early identification of persons at risk, e.g., by genetic or metabolic findings, an early application of specific training paradigms, non-invasive stimulation protocols, and/or pharmacological approaches targeting central adaptation and compensation may be suited to postpone the age at disease onset by years. Though ‘prevention is better than cure’, it may be possible to enhance mR in late life and in the late stages of disease, evidenced by studies that have shown positive effects of recent physical activity on adaptive capacities [114] and physical functioning in older adults [115] and in Parkinson’s Disease [116].

New training paradigms may comprise well-standardized protocols of cognitive and motor training, potentially boosted by non-invasive brain stimulation paradigms. As an innovative and holistic example, an evolutionary neuroscience perspective may inspire future behavioral interventions. This approach is aimed at challenging those domains which were most likely the key to foraging success of humans, for example by combining exercise with spatial navigation and memory tasks or by dual tasking during walking [117]. At the pharmacological level, neurotrophic factors such as BDNF as well as so-called resilience proteins may turn out to be therapeutic targets to maintain brain health in aging adults [92].

**Figure 5:**
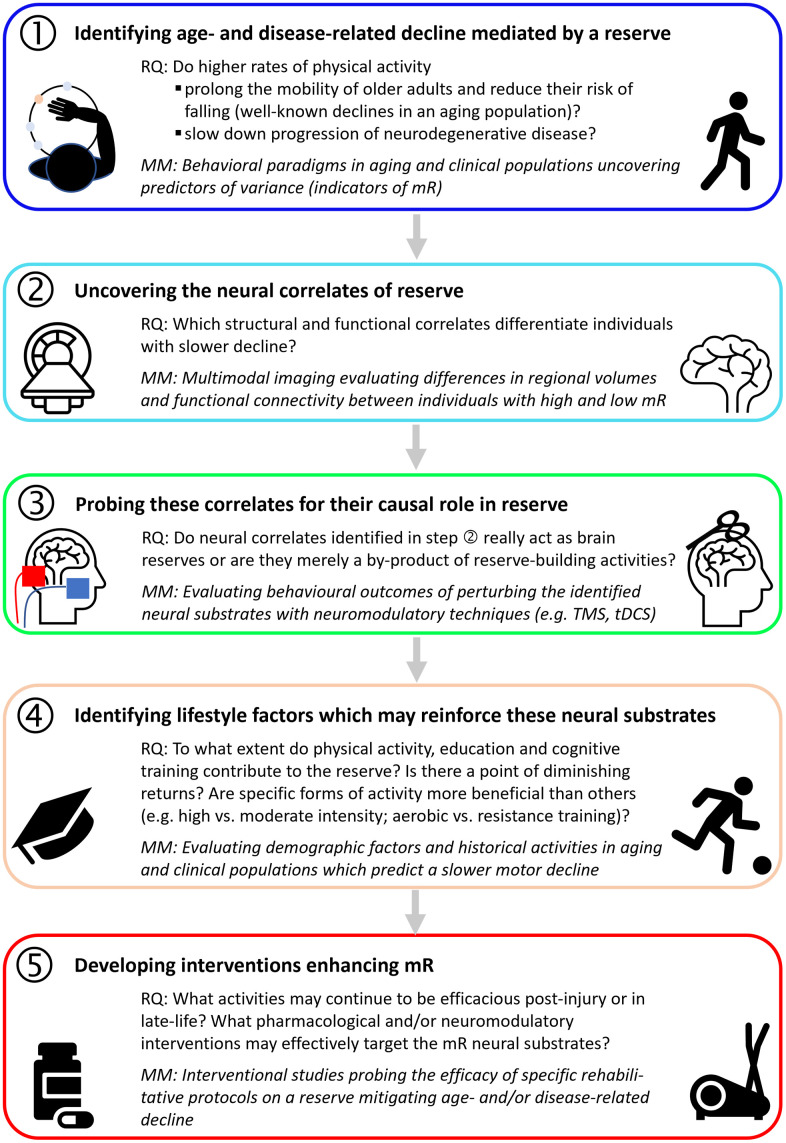
**A step-by-step approach to uncovering a motor reserve.** Each step is motivated by an outstanding research question (RQ), and possible materials and methods (MM) to search for answers are specified [images from stock art and flaticon (https://www.flaticon.com/free-icons/mri)].

But even irrespective of neurological conditions, specific enhancement of mR may be an efficacious contribution towards healthy aging (Figure 4). To get there, it will be essential to expedite our understanding of differences in individual motor resiliency towards ageing- and disease-related challenges and to develop the best possible estimate of mR. The motor reserve is indeed complex, with the intermingling of many different factors, such as genetic, environmental and lifestyle, that interact with the brain as it ages and in the face of injuries. While strong links have already been found regarding the effects of each of these factors on age and disease, it is necessary to build a comprehensive model of a motor reserve encompassing all the different factors. To address this challenge, we hereby propose a 5 steps-approach that will enable us to systematically uncover the best estimate of the motor reserve (Figure 5). This approach involves identifying robust behavioral measures and neural correlates of a reserve, experimental perturbation to establish causal relationships, and uncovering the influence of lifestyle factors. Ultimately, an estimate of mR will serve as an endpoint for studies on the efficacy of tailored treatment protocols in order to enhance the individual mR as early as possible (Figure 5).

## Supplementary Material

Supplementary Table 1
