# Supplementary material for: Considering the response in addition to the challenge – a narrative review in appraisal of a motor reserve framework
Source: Aging (Albany NY). 2024 Mar 18;16(6):5772–91. doi: 10.18632/aging.205667 (PMC11006496; doi:10.18632/aging.205667)
Supplement: Supplementary Table 1 [file aging-16-205667-s001.docx]

**Supplementary Table 1. Summary of articles containing the term “motor reserve”.**

| **Reference** | **Article type** | **Sample (sample size)** | **Aims/Objectives** | **Outcome measures** | **Key findings** |
| --- | --- | --- | --- | --- | --- |
| Hausdorff et al., 2008 | Research article | Healthy older adults (n=228) | Evaluate the influence of CR and MR as well as affect (e.g., anxiety, depressive symptoms) on dual tasking decrements (DTDs) in gait | Dual-task exercise (substractions and phoneme-monitoring)  Berg balance test and dynamic gait index | MR and CR influence DTD in healthy adults  Higher executive function correlate with lower gait variability during dual task |
| Palmer et al., 2009 | Research article | PD patients (n=10), healthy controls (n=10) | Investigating the relationship between compensation in PD and active motor reserve or newly recruited regions (NAR) | Novel area recruitment during force task in fMRI | In PD subjects, the activity within striato-thalamo-cortical and cerebello-thalamo-cortical regions at low speeds was similar to that in controls at higher speeds.  PD subjects off medication demonstrated NAR of bilateral cerebellum and primary motor cortex, which was partly normalized by levodopa. |
| Jouvent et al., 2016 | Research article | CADASIL patients (n=166) | Determining whether disability after stroke is related to the shape of the central sulcus, as a marker of the development of underlying motor connection | Shape of the central sulcus and disability | Shape of the central sulcus before stroke might predict post-stroke recovery, explaining individual variability in post-stroke disability |
| Sunwoo et al., 2017 | Research article | Drug-naïve PD patients (n=102) | Testing whether premorbid physical activity may enhance MR in PD | Premorbid exercise engagement  Motor function | Engagement in premorbid exercise acts as a proxy for an active reserve in the motor domain in patients with PD |
| Blume et al., 2017 | Research article | Patients with advanced stage PD (n=102) | Exploring the relationship of motor burden and educational attainment in patients with advanced stage PD | Educational attainment  UPDRS-III motor score | Inverse correlation between years of education and lower UPDRS -III motor score |
| Dalecki et al., 2019 | Research article | Sportspersons with concussion history (n=64), controls (n=62) | Examining factors that may influence skilled performance recovery | Performance in eye-hand coordination tasks | Individuals with concussion history and high training experience performed better.  Sport experience may provide more skill-related motor “reserve” despite concussion supoorting compensatory mechanisms |
| Olsson et al., 2020 | Research article | Long distance skiers (n=197,685), controls (n=197,684) | Investigating if physical activity is associated with long-term lower risk of PD and resilience against PD neuropathology before clinical manifestation | Incidence of PD during 21 years of follow-up | Skiers had lower incidence of PD than non-skiers  Results suggest the hypothesis of a motor reserve sustained by physical activity rather than neuroprotection in PD |
| Chung et al., 2020 | Research article | De novo PD patients (n=164) | Investigating the link between cognitive function and motor reserve in patients with newly diagnosed PD | Residual model of MR based on motor function and striatal dopamine depletion  Correlation analysis | Individual MR correlated with the years of education and verbal memory function  Higher MR estimates tended to be associated with a lower risk of dementia conversion |
| Chung et al., 2020 | Research article | De novo PD patients (n=205) | Exploring the functional brain network associated with motor reserve in early-stage PD | Residual model of mR based on motor function and striatal dopamine depletion | Identification of a MR network composed of the basal ganglia, inferior frontal cortex, insula, and cerebellar vermis  Functional connections within this network are associated with better resilience against PD-related pathologies |
| Chung et al., 2020 | Review article | n/a | Summarizing results on the concept of MR in PD | / | Premorbid experiences like physical activity and educationas well as modifiable factors, such as BMI and white matter intensity can enhance or reduce MR in PD, therefore modulating disease progression |
| Bede et al., 2021 | Perspective | n/a | Degenerative and regenerative processes in ALS | / | Discrepancy between the severity of radiological changes and limited functional impairment, suggests a degree of network redundancy, functional resilience or ‘motor reserve’ which should be explored. |
| Chung et al., 2021 | Research article | Drug-naïve PD patients (n=408) | Exploratory investigation of glucocerebrosidase (GBA) variants as potential determinants of motor reserve in PD | Residual model of MR in patients with vs. without GBA mutations | Detrimental effect of GBA variants on individual resilience against PD, with different impacts depending on the motor laterality. |
| Oh et al., 2022 | Research article | Drug-naïve PD patients (n=428) | Determining if cancer history can enhance motor reserve in PD | Motor scores and dopamine depletion (PET) in PD patients with vs. without malignancy | Groups with premorbid cancer showed lower UPDRS motor scores despite similar levels of dopamine depletion compared to those without neoplasia  History of cancer acts as a surrogate for motor reserve with a protective effect on PD |
| Kim et al., 2022 | Research article | Drug-naïve PD patients (n=238) | Exploring the white matter structural network associated with motor reserve in patients with newly diagnosed PD. | Residual model of mR, thres-hold-free network-based statistics (MRI) | Motor reserve-associated structural network identified mainly in the frontal region and cerebellum  Connectivity strength within this network indicates indicated maintained reserve with disease progression and affects the long-term motor prognosis of PD. |
| Chung et al., 2022 | Research article | Drug-naïve PD patients (n=163) | Investigating the link between cognitive function and MR in patients with newly diagnosed PD | Residual model of MR, cognitive composite scores, connectometry (MRI) | MR estimate correlated with verbal memory function and with white matter integrity in the left fornix in PD |
| Siciliano et al., 2022 | Research article | Patients with SCA2 (n=12) | Investigating MR and its association with both motor, cognitive performance and neural reserve in SCA2 patients | Motor Reserve Index Questionnaire (MRIq)  Functional connectivity (MRI) | MRIq measures correlate with the severity of motor symptoms, educational and intellectual levels, executive function, and processing speed  Correlation between MR estimate and functional connectivity in cerebral and cerebellar areas involved in specifically involved in motor functions |
| Bastos & Barbosa, 2022 | Review article | n/a | Review available evidence the relationship between physical activity and MR in neuropathologic and ageing context | / | Exercise strengthens MR  Motor learning, and not necessarily motor performance plays a critical role in mobilizing the benefits of exercise for MR |
| Jeong et al., 2022 | Research article | Drug-naïve PD patients (n=333) | Evaluate local striatal volume (LSV) as a neural correlate of MR in patients with early stage PD | Residual model of MR: Modelling of motor reserve estimate (MRE) as difference between the actual and predicted severity of motor symptoms  Local striatal volume (LSV, MRI) | LSV is crucial to MR in early stage PD and could be considered a neural correlate of MR  Significant positive correlation between LSV and mR estimates in the bilateral caudate, anterior putamen, and ventro-posterior putamen. |
| Hoenig et al., 2023 | Point of view | n/a | / | / | Further research on structural, functional, and molecular resilience signatures is warranted |
| Youn et al., 2023 | Research article | Drug-naïve PD patients (n=193) | Investigating brain structures relevant to the motor reserve in Parkinson’s disease (PD) | Residual model of MR: modelling of MR estimate (MRE) as difference between the actual and estimated severity of motor symptoms  Correlation analysis between MRE and DTI imaging features | MRE successfully predicted progression in patients with drug-naïve PD  Significant correlation between MRE and the activation of extra-basal ganglia brain regions (frontal lobe) |
| Pucci et al., 2023 | Research article | Healthy adults over 50  (n=75) | Evaluating MR as a flexible and dynamic construct that increases over time and compensates for age-related motor and cognitive loss | Motor reserve index Questionnaire (MRIq)  Current Physical Activity Questionnaire (CPAq)  Cognitive Reserve Index Questionnaire (CRIq)  Cognitive Function Dementia (CFD) | Significant relationship between age and MR  The MR effect is markedly higher in older adults compared to adults  MR and CRI are two independent types of reserve, both of which contribute in a different way to good cognitive functioning |

ALS, amyotrophic lateral sclerosis | CADASIL, cerebral autosomal dominant arteriopathy with subcortical infarcts and leukoencephalopathy | (f)MRI, (functional) magnetic resonance imaging | GBA, glucocerebrosidase | MR, motor reserve | n/a, not applicable | PD, Parkinson´s disease | PET, positron emission tomography | SCA2, spinocerebellar ataxia type 2.

By date of October 22, 2023, the fixed search term “motor reserve” yielded 27 hits on Pubmed. After exclusion of 6 articles (one about activity-dependent weakening, one about motor unit level, one about arm non-use, one mere computational article, and two errata), there remained 17 original articles and 4 review or perspective articles.
